# *XRCC1* Arg194Trp polymorphism, risk of nonmelanoma skin cancer and extramammary Paget’s disease in a Japanese population

**DOI:** 10.1007/s00403-012-1245-1

**Published:** 2012-05-26

**Authors:** Koji Chiyomaru, Tohru Nagano, Chikako Nishigori

**Affiliations:** Division of Dermatology, Department of Internal Related, Kobe University Graduate School of Medicine, 7-5-2 Kusunoki-cho, Chuo-ku, Kobe, 650-0017 Japan

**Keywords:** Skin cancer, SNP, *XRCC1*

## Abstract

The X-ray repair cross-complementing groups 1 gene plays an important role in base excision repair. At least three common single nucleotide polymorphisms frequently occur in this gene (Arg399Gln, Arg194Trp and Arg280His). Recent studies reported that these polymorphisms were associated with not only risk of visceral malignancy but also that of skin cancer such as basal cell carcinoma and squamous cell carcinoma, whereas the results of previous study vary among races. In this case–control study, we investigated whether these single nucleotide polymorphisms were associated with the risk of skin cancer in a Japanese population. The study population was composed of 197 patients with skin cancer (27 actinic keratoses, 47 basal cell carcinomas, 27 squamous cell carcinomas, 29 Bowen’s diseases, 46 malignant melanomas and 21 extramammary Paget’s diseases) and 93 control subjects. We genotyped two single nucleotide polymorphisms (Arg194Trp and Arg399Gln) using polymerase chain reaction-restriction fragments length polymorphism analysis. We found a significantly increased risk for basal cell carcinoma, squamous cell carcinoma and extramammary Paget’s disease associated with Arg194Trp [adjusted odds ratio (AOR) = 2.347, 3.587, 3.741, 95 % confidence interval (CI) 1.02–5.39, 1.19–10.8, 1.15–12.2, respectively]. We also found a significantly decreased risk for basal cell carcinoma associated with Gln399Gln (AOR = 0.259, 95 % CI 0.07–0.96). Our data suggest that the Arg194Trp polymorphism could be associated with nonmelanoma skin cancer and extramammary Paget’s disease risk in a Japanese population.

## Introduction

“Early detection, rapid cure” is most effective strategy for cancer treatment. The outcome from educational campaigns by oncologists has not been enough for reducing the number of patients with advanced cancer. Furthermore, the cost of medical expense is growing with each passing year. Detection of high risk population for cancer seems to become a practical way for educating people. For example, detecting a DNA variation associated with cancer susceptibility of each individual could become an effective strategy for solving the problem.

Cancers are not single-gene diseases. Many oncogenes, tumor suppressor genes and DNA repair genes are all likely to be involved. DNA in most cells is regularly damaged by endogenous and exogenous mutagens. Unrepaired damage can result in apoptosis or may lead to unregulated cell growth and cancer. Inheritance of genetic variants at one or more loci results in reduced DNA repair capacity. DNA repair enzymes have been reported to play an important role in the carcinogenesis of several cancers. DNA repair enzymes are involved in repairing damaged DNA and at least four pathways operate on specific types of DNA damage: the base excision repair (BER), nucleotide excision repair (NER), double strand break repair (DSBR) and mismatch repair (MMR) pathways. Enzymes involved in BER include XRCC1, those involved in NER include XPC, XPD and ERCC1, those involved in DSBR include BRCA1, BRCA2 and XRCC3, and those involved in MMR include MLH1, MSH2, PMS2 and MSH6 [9].

From this standpoint, we focused on single nucleotide polymorphisms (SNPs) of the DNA repair gene, X-ray repair cross-complementing groups 1 (*XRCC1*) which was the first identified human gene involved in the repair of single strand break (SSB) of DNA formed by exposure to ionizing radiation and alkylating agents [20]. The SNPs may alter the function and capacity of DNA repair. Although enzymatic activity has not been revealed yet, *XRCC1* interacts with many proteins involved in base excision repair (BER) and SSB repair. It has been supposed that *XRCC1* plays a role as a scaffold protein for coordinating and facilitating the various DNA repair pathways [11, 13]. The SNPs of *XRCC1* has been associated with a risk factor of cancers such as lung cancer [3], breast cancer [15], prostate cancer [7, 19], and also with skin cancers [6, 9, 17].

In the case of skin cancer, most people can see the lesion by his or her own eyes. So the information about his/her susceptibility to skin cancer enables him/her to realize “Early detection and rapid cure”.

On the other hand, a contribution of SNPs to skin cancer has ethnic and racial differences. In that way, further insight into each society is important.

In the present study, we investigated the association between polymorphisms of *XRCC1* and skin cancer in a Japanese population.

## Materials and methods

### Study population

One-hundred and ninety-seven patients (mean age ± standard deviation (SD), 72.9 ± 12.2 years; age range 35–95 years) with skin cancer [27 actinic keratoses (AK), 47 basal cell carcinomas (BCC), 27 squamous cell carcinomas (SCC), 29 Bowen’s disease (BD), 46 malignant melanomas (MM) and 21 extramammary Paget’s diseases (EPD)] and 93 control subjects (mean age ± SD, 66.1 ± 13.7 years; age range 28–89) who visited the dermatology clinic in Kobe University Hospital between April 2004 and November 2010 were enrolled in this study. Clinical diagnosis was confirmed by histopathological analysis in all cases.

We subcategorized the patients according to the location of cancer lesions whether on sun exposure site (head, neck and hand) or not. The frequencies of BCC were: sun exposure site, 89.4 % and not sun exposure site, 10.6 %. Those of SCC were: sun exposure site, 51.9 % and not sun exposure site, 48.1 %. Those of MM were: sun exposure site, 17.4 % and not sun exposure site, 82.6 %.

The control group consists of 93 age- and sex-matched non-affected unrelated Japanese individuals from the same area in Japan with minor fungi, bacterial infections or seborrheic keratosis. We excluded the patients with other malignant disorders (Table 1).

**Table 1:** Clinical characteristics and numbers among cases and controls

Characteristics	Cases (with AK diagnosis), No. (%) (*n* = 27)	Cases (with BCC diagnosis), No. (%) (*n* = 47)	Cases (with SCC diagnosis), No. (%) (*n* = 27)	Cases (with BD diagnosis), No. (%) (*n* = 29)	Cases (with MM diagnosis), No. (%) (*n* = 46)	Cases (with EPD diagnosis), No. (%) (*n* = 21)	Controls, No. (%) (*n* = 93)
Men	8 (29.6)	25 (53.2)	14 (51.9)	11 (37.9)	24 (52.2)	11 (52.4)	48 (51.6)
Women	19 (70.4)	22 (46.8)	13 (48.1)	18 (62.1)	22 (47.8)	10 (47.6)	45 (48.4)

*AK* actinic keratosis, *BD* bowen’s disease, *BCC* basal cell carcinoma, *SCC* squamous cell carcinoma, *MM* malignant melanoma, *EPD* extramammary Paget’s disease

We handled these patients as anonymous samples about which nobody could know their personal information other than their genotypes. Written informed consent was also obtained from all participants. The Medical Ethics Committee of Kobe University approved this work that was conducted in accordance with the Declaration of Helsinki principles.

### Genetics analysis

Genomic DNA was extracted from peripheral blood using Qiagen FlexiGene DNA kit. The nucleotide changes were determined by polymerase chain reaction-restriction fragments length polymorphism (PCR-RFLP) analysis using the isolated genomic DNA as a template. PCR for the *XRCC1* Arg194Trp (C > T) polymorphism was carried out using primers 5′ GCC CCG TCC CAG GTA 3′ and 5′ AGC CCC AAG ACC CTT TCA CT 3′. DNA was amplified for 35 cycles comprising denaturing at 94° C for 60 s, annealing at 61° C for 60 s and extension at 72° C for 60 s. The PCR products were detected after incubation with the restriction endonuclease *Pvu*II at 37° C for 2 h.

The forward and reverse primers for the *XRCC1* Arg399Gln (G > A) polymorphism used were 5′ CCC CAA GTA CAG CCA GGT C 3′ and 5′ TGT CCC GCT CCT CTC AGT AG 3′, respectively. PCRs were run for 35 cycles: 94° C for 60 s, 62° C for 60 s, 72° C for 60 s, and products were digested with the restriction endonuclease *Msp*I at 37° C for 2 h.

### Statistical analysis

For statistical analysis, we used SPSS for Windows, version 17.0 (SPSS Japan Inc.) to calculate the crude odds ratio (OR), the adjusted odds ratio (AOR) and 95 % confidence interval (CI). All adjusted models included age and sex.

## Results

### Polymorphisms of *XRCC1* codons 194 and 399 and skin cancer risk

The *XRCC1* gene polymorphisms Arg194Trp and Arg399Gln were investigated. Their genotype distributions in skin cancers and controls are shown in Table 2. We found a significantly increased risk for skin cancers associated with Arg194Trp (AOR = 1.810, 95 % CI 1.03–3.18).

**Table 2:** Crude OR, adjusted OR and 95 % CI of the XRCC1 polymorphisms Arg194Trp and Arg399Gln among cases and controls

Genotypes	Cases (with SC^a^ diagnosis), No. (%) (*n* = 197)	Controls, No. (%) (*n* = 93)	Crude OR (95 % CI)	Adjusted OR^b^ (95 % CI)	Cases (with NMSC^c^ diagnosis), No. (%) (*n* = 74)	Controls, No. (%) (*n* = 93)	Crude OR (95 % CI)	Adjusted OR^b^ (95 % CI)
Codon 194								
Arg/Arg	93 (47.2)	52 (55.9)	1.0 (Reference)	1.0 (Reference)	34 (45.9)	52 (55.9)	1.0 (Reference)	1.0 (Reference)
Arg/Trp	84 (42.6)	28 (30.1)	1.677 (0.97–2.90)	1.810 (1.03–3.18)	33 (44.6)	28 (30.1)	1.804 (0.93–3.49)	2.380 (1.15–4.95)
Trp/Trp	20 (10.2)	13 (14.0)	0.860 (0.40–1.87)	0.877 (0.40–1.94)	7 (9.5)	13 (14.0)	0.914 (0.34–2.44)	1.092 (0.38–3.11)
Arg/Trp + Trp/Trp	104 (52.8)	41 (44.1)	1.418 (0.86–2.33)	1.517 (0.91–2.54)	40 (54.1)	41 (44.1)	1.522 (0.83–2.79)	1.955 (1.00–3.81)
Codon 399								
Arg/Arg	111 (56.3)	51 (54.8)	1.0 (Reference)	1.0 (Reference)	47 (63.5)	51 (54.8)	1.0 (Reference)	1.0 (Reference)
Arg/Gln	61 (31.0)	21 (22.6)	1.335 (0.74–2.42)	1.387 (0.75–2.56)	21 (28.4)	21 (22.6)	1.090 (0.53–2.23)	0.927 (0.44–1.98)
Gln/Gln	25 (12.7)	21 (22.6)	0.547 (0.28–1.07)	0.590 (0.29–1.20)	6 (8.1)	21 (22.6)	0.297 (0.11–0.80)	0.318 (0.11–0.92)
Arg/Gln + Gln/Gln	86 (43.7)	42 (45.2)	0.941 (0.57–1.55)	1.000 (0.60–1.67)	27 (36.5)	42 (45.2)	0.694 (0.37–1.29)	0.632 (0.33–1.23)

*OR* odds ratio, *CI* confidence interval, *SC* skin cancer, *NMSC* nonmelanoma skin cancer

^a^SC includes actinic keratosis, Bowen’s disease, basal cell carcinoma, squamous cell carcinoma, malignant melanoma and extramammary Paget’s disease

^b^Adjusted for age and sex

^c^NMSC includes basal cell carcinoma and squamous cell carcinoma

### Polymorphisms of *XRCC1* codons 194 and 399 and each category of skin cancer risk

We investigated the *XRCC1* gene polymorphisms of codon 194 and 399 in the each skin cancer (Table 3). We found a significantly increased risk for BCC, SCC and EPD associated with Arg194Trp (AOR = 2.347, 3.587, 3.741, 95 % CI 1.02–5.39, 1.19–10.8, 1.15–12.2, respectively). We also found a significantly decreased risk for BCC associated with Gln399Gln (AOR = 0.259, 95 % CI 0.07–0.96).

**Table 3:** Crude OR, adjusted OR and 95 % CI of the XRCC1 polymorphisms Arg194Trp and Arg399Gln among cases and controls

Genotypes	Cases (with AK diagnosis), No. (%) (*n* = 27)	Controls, No. (%) (*n* = 93)	Crude OR (95 % CI)	Adjusted OR^a^ (95 % CI)	Cases (with BCC diagnosis), No. (%) (*n* = 47)	Controls, No. (%) (*n* = 93)	Crude OR (95 % CI)	Adjusted OR^a^ (95 % CI)
Codon 194								
Arg/Arg	16 (59.3)	52 (55.9)	1.0 (Reference)	1.0 (Reference)	21 (44.7)	52 (55.9)	1.0 (Reference)	1.0 (Reference)
Arg/Trp	10 (37.0)	28 (30.1)	1.161 (0.47–2.90)	1.331 (0.47–3.77)	20 (42.6)	28 (30.1)	1.769 (0.82–3.80)	2.347 (1.02–5.39)
Trp/Trp	1 (3.7)	13 (14.0)	0.250 (0.03–2.06)	0.315 (0.04–2.74)	6 (12.8)	13 (14.0)	1.143 (0.38–3.41)	1.315 (0.42–4.09)
Arg/Trp + Trp/Trp	11 (40.7)	41 (44.1)	0.872 (0.37–2.08)	0.989 (0.38–2.60)	26 (55.3)	41 (44.1)	1.570 (0.78–3.18)	1.957 (0.92–4.16)
Codon 399								
Arg/Arg	13 (48.1)	51 (54.8)	1.0 (Reference)	1.0 (Reference)	32 (68.1)	51 (54.8)	1.0 (Reference)	1.0 (Reference)
Arg/Gln	10 (37.0)	21 (22.6)	1.868 (0.71–4.92)	1.566 (0.55–4.46)	12 (25.5)	21 (22.6)	0.911 (0.40–2.10)	0.734 (0.31–1.77)
Gln/Gln	4 (14.8)	21 (22.6)	0.747 (0.22–2.56)	0.842 (0.20–3.61)	3 (6.4)	21 (22.6)	0.228 (0.06–0.83)	0.259 (0.07–0.96)
Arg/Gln + Gln/Gln	14 (51.9)	42 (45.2)	1.308 (0.55–3.09)	1.256 (0.49–3.21)	15 (31.9)	42 (45.2)	0.569 (0.27–1.19)	0.514 (0.24–1.11)

Genotypes	Cases (with SCC diagnosis), No. (%) (*n* = 27)	Controls, No. (%) (*n* = 93)	Crude OR (95 % CI)	Adjusted OR^a^ (95 % CI)	Cases (with BD diagnosis), No. (%) (*n* = 29)	Controls, No. (%) (*n* = 93)	Crude OR (95 % CI)	Adjusted OR^a^ (95 % CI)
Codon 194								
Arg/Arg	13 (48.1)	52 (55.9)	1.0 (Reference)	1.0 (Reference)	14 (48.3)	52 (55.9)	1.0 (Reference)	1.0 (Reference)
Arg/Trp	13 (48.1)	28 (30.1)	1.857 (0.76–4.55)	3.587 (1.19–10.8)	11 (37.9)	28 (30.1)	1.459 (0.59–3.64)	1.835 (0.69–4.87)
Trp/Trp	1 (3.7)	13 (14.0)	0.308 (0.04–2.57)	0.295 (0.03–3.15)	4 (13.8)	13 (14.0)	1.143 (0.32–4.06)	1.395 (0.37–5.29)
Arg/Trp + Trp/Trp	14 (51.9)	41 (44.1)	1.366 (0.58–3.22)	2.251 (0.83–6.14)	15 (51.7)	41 (44.1)	1.359 (0.59–3.13)	1.685 (0.70–4.07)
Codon 399								
Arg/Arg	15 (55.6)	51 (54.8)	1.0 (Reference)	1.0 (Reference)	14 (48.3)	51 (54.8)	1.0 (Reference)	1.0 (Reference)
Arg/Gln	9 (33.3)	21 (22.6)	1.457 (0.55–3.84)	1.199 (0.42–3.41)	10 (34.5)	21 (22.6)	1.735 (0.67–4.52)	1.623 (0.61–4.30)
Gln/Gln	3 (11.1)	21 (22.6)	0.486 (0.13–1.85)	0.556 (0.12–2.63)	5 (17.2)	21 (22.6)	0.867 (0.28–2.71)	1.011 (0.30–3.36)
Arg/Gln + Gln/Gln	12 (44.4)	42 (45.2)	0.971 (0.41–2.30)	0.928 (0.36–2.41)	15 (51.7)	42 (45.2)	1.301 (0.56–3.00)	1.293 (0.55–3.05)

Genotypes	Cases (with MM diagnosis), No. (%) (*n* = 46)	Controls, No. (%) (*n* = 93)	Crude OR (95 % CI)	Adjusted OR^a^ (95 % CI)	Cases (with EPD diagnosis), No. (%) (*n* = 21)	Controls, No. (%) (*n* = 93)	OR (95 % CI)	Adjusted OR^a^ (95 % CI)
Codon 194								
Arg/Arg	21 (45.7)	52 (55.9)	1.0 (Reference)	1.0 (Reference)	8 (38.1)	52 (55.9)	1.0 (Reference)	1.0 (Reference)
Arg/Trp	20 (43.5)	28 (30.1)	1.769 (0.82–3.80)	1.718 (0.79–3.72)	10 (47.6)	28 (30.1)	2.321 (0.82–6.55)	3.741 (1.15–12.2)
Trp/Trp	5 (10.9)	13 (14.0)	0.952 (0.30–3.01)	0.981 (0.30–3.19)	3 (14.3)	13 (14.0)	1.500 (0.35–6.46)	1.454 (0.28–7.51)
Arg/Trp + Trp/Trp	25 (54.3)	41 (44.1)	1.510 (0.74–3.07)	1.486 (0.73–3.03)	13 (61.9)	41 (44.1)	2.061 (0.78–5.44)	2.884 (0.98–8.46)
Codon 399								
Arg/Arg	26 (56.5)	51 (54.8)	1.0 (Reference)	1.0 (Reference)	11 (52.4)	51 (54.8)	1.0 (Reference)	1.0 (Reference)
Arg/Gln	13 (28.3)	21 (22.6)	1.214 (0.53–2.81)	1.209 (0.52–2.81)	7 (33.3)	21 (22.6)	1.545 (0.53–4.53)	1.375 (0.44–4.32)
Gln/Gln	7 (15.2)	21 (22.6)	0.654 (0.25–1.74)	0.659 (0.25–1.77)	3 (14.3)	21 (22.6)	0.662 (0.17–2.62)	1.027 (0.21–5.08)
Arg/Gln + Gln/Gln	20 (43.5)	42 (45.2)	0.934 (0.46–1.90)	0.909 (0.44–1.86)	10 (47.6)	42 (45.2)	1.104 (0.43–2.85)	1.180 (0.42–3.30)

*OR* odds ratio, *CI* confidence interval, *AK* actinic keratosis, *BD* bowen’s disease, *BCC* basal cell carcinoma, *SCC* squamous cell carcinoma, *MM* malignant melanoma, *EPD* extramammary Paget’s disease

^a^Adjusted for age and sex

### Polymorphisms of *XRCC1* codons 194 and 399 and nonmelanoma skin cancer (NMSC) risk

Next we analyzed relationship between *XRCC1* SNP and NMSC (BCC and SCC) (Table 2). We found a significantly increased risk for NMSC associated with Arg194Trp (AOR = 2.380, 95 % CI 1.15–4.95). We found a significantly decreased risk for NMSC associated with Gln399Gln (AOR = 0.318, 95 % CI 0.11–0.92).

## Discussion

In our present study, although our statistical analysis of each skin cancer and controls had potential biases, a significantly increased risk for skin cancer was associated with the SNP of *XRCC1* Arg194Trp (AOR = 1.810, 95 % CI 1.03–318) (Table 2). Two studies [6, 9] reported an association of risk in skin cancer with *XRCC1* Arg194Trp, but the results were controversial. Han et al. reported the increased risk of SCC with Arg194Trp, on the other hand, Kang et al. reported the decreased risk of SCC with the same SNP. These results indicate that the risk of skin cancer in SNP in *XRCC1* varies among races. In our present study, the polymorphism of *XRCC1* Arg194Trp has been shown to be an important factor in susceptibility to skin cancer among Japanese population.

Association of *XRCC1* Arg194Trp could be not only with MNSC but also with EPD (Tables 2, 3). Although the number of each skin cancer has still been small, the polymorphisms of *XRCC1* Arg194Trp could be involved in carcinogenesis of BCC and SCC. On the other hand, in the case of AK and BD, *XRCC1* Arg194Trp has no statistical significance, which indicates *XRCC1* Arg194Trp would be related to susceptibility to not in situ but advanced skin cancer. In addition, actinic keratosis usually heralds SCC. Many of SCC cases should also have AKs, thus, it could be a useful indicator for the progression of the disease. Little is known about the molecular mechanisms or genetic disorder of pathogenesis of EPD. It was reported that there were high expressions of c-erb B2 and PIG7/LITAF in EPD [12, 18], and Ki-67 and cycline D1 in invasive EPD [1]. There is no report investigating the *XRCC1* Arg194Trp polymorphism in EPD. Since EPD occurs in sun-protected areas, the true meaning of the SNPs in our patients needs to be analyzed in a larger number of patients and in relation to other genetic aberrations. As far as we know, our result is a new indication for the relationship between polymorphism of *XRCC1* and EPD.

In the analysis of NMSC on the polymorphisms of *XRCC1*, we found a significantly increased risk with Arg194Trp (AOR = 2.380, 95 % CI 1.15–4.95) and also decreased risk with Gln399Gln (AOR = 0.318, 95 % CI 0.11–0.92). Previous reports showed that *XRCC1* Gln399Gln homozygote variant was associated with a significantly reduced risk of NMSC [17], which supported our results.

The inverse association of different loci (codon 194 and 399) of *XRCC1* has been observed in previous reports [6, 9]. The reason for this still remains unknown, several factors relevant to this polymorphism, such as different populations, different skin types [4] and differently damaged levels of apoptotic mechanisms, might cause different outcomes. Our data suggest that the Arg194Trp and Arg399Gln polymorphisms may be differently associated with NMSC in a Japanese population.

In the case of skin carcinogenesis, ultraviolet (UV) plays an important role [5], especially in the induction of mutations in both oncogenes and tumor suppressor genes [2, 8]. It has been reported that UV has been implicated in inhibition of anti-tumor T-cell immune response through elevated interleukin 10 (IL-10) and other cytokines level [21]. Recently, we have shown that IL-10 polymorphisms are strongly correlated to sun-related skin cancers in a Japanese population [16]. Therefore, we thought that under the circumstances without UV effects, BER deficiency could be more involved in carcinogenesis in the skin. However, further analysis of our data indicated that polymorphism of *XRCC1* codon 399 and 194 revealed much more significantly increased risk for BCC on sun-exposed area associated with Arg194Trp and Arg194Trp + Trp194Trp (AOR = 3.304, 95 % CI 1.36–8.03 and AOR = 2.643, 95 % CI 1.19–5.89, respectively) (Table 4). Although the case numbers are low in this study and thus, the statistical power is low, our results indicate a possibility of association of the polymorphism of *XRCC1* with UV-induced carcinogenesis. Actually, Moser et al. [14] indicated the association of *XRCC1* with not only BER but also with nucleotide excision repair pathway which has a major role in repairing UV-induced DNA damage.

**Table 4:** Crude OR, adjusted OR and 95 % CI of the XRCC1 polymorphisms Arg194Trp and Arg399Gln among cases and controls

Genotypes	Cases (with BCC diagnosis), No. (%) (*n* = 47)	Controls, No. (%) (*n* = 93)	Crude OR (95 % CI)	Adjusted OR^a^ (95 % CI)	Cases (with SCC diagnosis), No. (%) (*n* = 27)	Controls, No. (%) (*n* = 93)	Crude OR (95 % CI)	Adjusted OR^a^ (95 % CI)
Sun exposure site									
Codon 194									
Arg/Arg	16 (38.1)	52 (55.9)	1.0 (Reference)	1.0 (Reference)	7 (50.0)	52 (55.9)	1.0 (Reference)	1.0 (Reference)
Arg/Trp	20 (47.6)	28 (30.1)	2.321 (1.04–5.18)	3.304 (1.36–8.03)	6 (42.9)	28 (30.1)	1.592 (0.49–5.20)	3.928 (0.86–17.9)
Trp/Trp	6 (14.3)	13 (14.0)	1.500 (0.49–4.59)	1.749 (0.54–5.62)	1 (7.1)	13 (14.0)	0.571 (0.06–5.06)	0.420 (0.03–6.04)
Arg/Trp + Trp/Trp	26 (61.9)	41 (44.1)	2.061 (0.98–4.34)	2.643 (1.19–5.89)	7 (50.0)	41 (44.1)	1.268 (0.41–3.91)	2.292 (0.61–8.69)
Codon 399									
Arg/Arg	30 (71.4)	51 (54.8)	1.0 (Reference)	1.0 (Reference)	8 (57.1)	51 (54.8)	1.0 (Reference)	1.0 (Reference)
Arg/Gln	9 (21.4)	21 (22.6)	0.729 (0.30–1.80)	0.594 (0.23–1.53)	4 (28.6)	21 (22.6)	1.214 (0.33–4.47)	1.191 (0.29–4.97)
Gln/Gln	3 (7.1)	21 (22.6)	0.243 (0.07–0.88)	0.277 (0.07–1.03)	2 (14.3)	21 (22.6)	0.607 (0.12–3.10)	1.139 (0.12–10.7)
Arg/Gln + Gln/Gln	12 (28.6)	42 (45.2)	0.486 (0.22–1.06)	0.447 (0.20–1.01)	6 (42.9)	42 (45.2)	0.911 (0.29–2.83)	1.124 (0.31–4.09)
Not sun exposure site									
Codon 194									
Arg/Arg	5 (100)	52 (55.9)	1.0 (Reference)	1.0 (Reference)	6 (46.2)	52 (55.9)	1.0 (Reference)	1.0 (Reference)
Arg/Trp	0 (0.0)	28 (30.1)	–	–	7 (53.8)	28 (30.1)	2.167 (0.66–7.07)	3.664 (0.96–13.9)
Trp/Trp	0 (0.0)	13 (14.0)	–		0 (0.0)	13 (14.0)	–	–
Arg/Trp + Trp/Trp	0 (0.0)	41 (44.1)	–	–	7 (53.8)	41 (44.1)	1.268 (0.41–3.91)	2.292 (0.61–8.69)
Codon 399									
Arg/Arg	2 (40.0)	51 (54.8)	1.0 (Reference)	1.0 (Reference)	7 (53.8)	51 (54.8)	1.0 (Reference)	1.0 (Reference)
Arg/Gln	3 (60.0)	21 (22.6)	3.643 (0.57–23.4)	2.567 (0.36–18.2)	5 (38.5)	21 (22.6)	1.735 (0.49–6.09)	1.272 (0.34–4.78)
Gln/Gln	0 (0.0)	21 (22.6)	–	–	1 (7.7)	21 (22.6)	0.347 (0.04–3.00)	0.384 (0.04–3.45)
Arg/Gln + Gln/Gln	3 (60.0)	42 (45.2)	1.821 (0.29–11.4)	1.573 (0.23–10.1)	6 (46.2)	42 (45.2)	1.041 (0.33–3.34)	0.883 (0.26–2.98)

Genotypes	Cases **(**with MM diagnosis), No. (%) (*n* = 46)	Controls, No. (%) (*n* = 93)	Crude OR (95 % CI)	Adjusted OR^a^ (95 % CI)				
Sun exposure site									
Codon 194									
Arg/Arg	6 (75.0)	52 (55.9)	1.0 (Reference)	1.0 (Reference)				
Arg/Trp	2 (25.0)	28 (30.1)	0.619 (0.12–3.27)	0.533 (0.10–2.93)				
Trp/Trp	0 (0.0)	13 (14.0)	–	–				
Arg/Trp + Trp/Trp	2 (25.0)	41 (44.1)	0.423 (0.08–2.21)	0.374 (0.07–2.02)				
Codon 399									
Arg/Arg	3 (37.5)	51 (54.8)	1.0 (Reference)	1.0 (Reference)				
Arg/Gln	2 (25.0)	21 (22.6)	1.619 (0.25–10.4)	1.574 (0.24–10.3)				
Gln/Gln	3 (37.5)	21 (22.6)	2.429 (0.45–13.0)	2.347 (0.43–12.9)				
Arg/Gln + Gln/Gln	5 (62.5)	42 (45.2)	2.024 (0.46–8.97)	2.041 (0.46–9.14)				
Not sun exposure site									
Codon 194									
Arg/Arg	15 (39.5)	52 (55.9)	1.0 (Reference)	1.0 (Reference)				
Arg/Trp	18 (47.4)	28 (30.1)	2.229 (0.98–5.09)	2.161 (0.94–4.97)				
Trp/Trp	5 (13.2)	13 (14.0)	1.333 (0.41–4.34)	1.340 (0.40–4.47)				
Arg/Trp + Trp/Trp	23 (60.5)	41 (44.1)	1.945 (0.90–4.19)	1.907 (0.88–4.13)				
Codon 399									
Arg/Arg	23 (60.5)	51 (54.8)	1.0 (Reference)	1.0 (Reference)				
Arg/Gln	11 (28.9)	21 (22.6)	1.161 (0.48–2.80)	1.160 (0.48–2.82)				
Gln/Gln	4 (10.5)	21 (22.6)	0.422 (0.13–1.37)	0.430 (0.13–1.40)				
Arg/Gln + Gln/Gln	15 (39.5)	42 (45.2)	0.792 (0.37–1.71)	0.782 (0.36–1.69)				

*OR* odds ratio, *CI* confidence interval, *BCC* basal cell carcinoma, *SCC* squamous cell carcinoma, *MM* malignant melanoma

^a^Adjusted for age and sex

The functional significance of SNPs is still largely unknown. Recent reports show that the SNPs of *XRCC1* have been focused on not only in silico analysis but also on in vitro and in vivo [10]. Further functional analyses of the SNPs are needed.

Being different from visceral cancers, almost all skin cancers are visible. Thus, early detection of skin anomaly should be possible for not only medical doctors but also for each individual. Nevertheless, there are still many patients with advanced skin cancer. Thus, screening of skin cancer risk for each individual enables them to know the importance of careful examination on their own skin in their daily life, leading to reduction in both the number of patients with advanced skin cancer and the cost of medical expense.

In conclusion, *XRCC1* gene polymorphisms, the Arg194Trp genotype increased the risk of NMSC and EPD in a Japanese population. Consequently, this genotype could be used as biomarkers for the estimation of skin cancer risk, leading to education to the public, enabling the risk-individuals to obtain “Early detection and rapid cure”.
